# Transition Readiness in Pediatric Chronic Digestive Diseases: A Regional Perspective from North-Eastern Romania

**DOI:** 10.3390/medicina60122104

**Published:** 2024-12-22

**Authors:** Silvia Cristina Poamaneagra, Felicia Galos, Elena Tataranu, Catalina Mihai, Carmen Anton, Cristiana-Mihaela Andronic, Georgiana-Emmanuela Gilca-Blanariu, Gheorghe G. Balan, Oana Timofte, Liliana Anchidin-Norocel, Oana Maria Rosu, Smaranda Diaconescu

**Affiliations:** 1 Doctoral School, George Emil Palade University of Medicine, Pharmacy, Science and Technology, 540139 Targu Mures, Romania; silviastrat89@yahoo.ro (S.C.P.); oana.rosu@umfst.ro (O.M.R.); 2 Marie Curie Emergency Childrens Hospital, 077120 Bucharest, Romania; 3 Faculty of Medicine, Carol Davila University of Medicine and Pharmacy, 050474 Bucharest, Romania; 4 Clinical Department of Pediatrics, Sf. Ioan cel Nou, Emergency Hospital, 720224 Suceava, Romania; 5 Faculty of Medicine, “Grigore T. Popa” University of Medicine and Pharmacy, 700115 Iasi, Romania; catalinamihai@yahoo.com (C.M.); carmen.anton@umfiasi.ro (C.A.); andronic_mihaela-cristiana@d.umfiasi.ro (C.-M.A.); georgiana.gilca@gmail.com (G.-E.G.-B.); balan.gheo@me.com (G.G.B.); oana.timofte@umfiasi.ro (O.T.); 6 Department of Gastroenterology and Hepatology, “St. Spiridon” Emergency Hospital, 700111 Iasi, Romania; 7 Faculty of Medicine and Biological Sciences, Stefan cel Mare University of Suceava, 720229 Suceava, Romania; liliana.norocel@usm.ro; 8 Faculty of Medicine, “Titu Maiorescu” University of Medicine, 050474 Bucharest, Romania; smaranda.diaconescu@prof.utm.ro

**Keywords:** transition, pediatric chronic digestive diseases, transition readiness, potentially modifiable risk factors

## Abstract

*Background*: The transition from the pediatric to the adult healthcare system is a challenging process involving adolescents, parents, and pediatric and adult specialists. For a successful approach for an organized transition program, we must assess the level of training of adolescents facing transition. *Methods*: We applied a clinic-based questionnaire measuring perceived self-management skills, adherence to health-related tasks, medication knowledge, and social adjustment to pediatric patients with chronic digestive diseases from North-East Romanian medical units, including a tertiary center and private practice offices. *Results*: There were 124 participants; 73.38% from rural areas, 26.62% from urban areas; 59.67% were females, and 40.33% were males; 91.93% attended school and 73.4% declared wanting to pursue university classes after turning 18. Adolescents from urban areas showed better medication managerial (*p* < 0.01) and tracking healthcare change skills. Significant correlations were found between medication and appointment making, tracking health dynamics, and communication skills. Other correlations were found between communication skills and medication knowledge, appointments management, and tracking healthcare dynamics. All the investigated domains were positively correlated with the overall scores, highlighting the potential impact of active targeted interventions during transition. *Conclusions*: We identified significant areas to address and potentially influence during an organized transition program such as communication skills and knowledge regarding the chronic disease and the followed medication.

## 1. Introduction

In the last years there has been major progress in medicine and healthcare policies, facilitating easier access to screening programs leading to early detection of chronic diseases, enabling complex evaluation in multidisciplinary medical teams and long-term modern treatment options. It is estimated that more than 90% of children with chronic disease will survive into adulthood, presenting challenges for healthcare providers in preparing adolescents and young adults for the transition to adult medicine [1,2,3,4,5].

With the increase in survival of children and adolescents with chronic diseases such as congenital heart disease, cystic fibrosis, congenital hepatobiliary diseases, neoplasms, the prevalence of adolescents requiring a transition from pediatric to adult services has increased considerably. Also, in the last decade, international scientific interest on this topic has increased and various transition service models have been implemented in different countries [6].

The challenge regarding the transition process consists in the fact that the pediatric healthcare system is family-oriented, and it is based to a fairly large percentage on the involvement and decisions of the parents, whereas the adult healthcare system is patient-specific and requires a certain level of independence in decision making [7].

Proposed transition models over time have evolved from initially placing responsibility for the process on the pediatric specialist to a shared responsibility between various pediatric and adult healthcare professionals (e.g., doctors, nurses, social workers, psychologists) [8].

Despite all the efforts, it appears that more than half of American adolescents suffering from chronic diseases are not prepared for the transition process or have inadequate support during this period. The authors tried to identify the stressors and risk factors that make the transition process difficult, to determine which of them can be addressed and modified and, eventually, to establish strategies by which these factors can be addressed at a population level [9].

Up to this moment, in Romania there is no standardized national transition protocol for adolescents with chronic diseases, transfer occurring at the age of 18 without an investigation of the presence or absence of necessary skills for self-managing the chronic condition and without any active intervention to facilitate this process. In the absence of a formal transition protocol, our teenager patients are often lost in the system, creating a critical gap that must be bridged.

This study aimed to evaluate the key aspects of the transition process for adolescents with chronic digestive diseases, focusing on their preparedness for managing their healthcare as they transition from pediatric to adult care. Specifically, the study assessed their awareness of the importance of self-management, examined their desire to be involved in healthcare decisions, evaluated their knowledge of prescribed medications and their side effects, and assessed their skills in managing medication and medical appointments. Additionally, the study investigated their ability to monitor and report changes in their health, evaluate their communication skills, and assess their overall transition readiness. It also identified non-modifiable and potentially modifiable risk factors that can be addressed during this critical transition period.

## 2. Materials and Methods

We employed a cross-sectional study using the survey methodology including the number of 124 children aged between 14 and 17 with chronic digestive diseases from a tertiary center in North Eastern Romania and from private practice offices.

The study was approved by decision no. 38761/09.11.2020 of the Research and Ethics Commission of ‘St. Mary’ Hospital. All patients included in the study agreed to participate by having their informed consent form signed by themselves and their parents or legal guardians. Patient data were processed and classified according to international research guidelines [10]. Inclusion and exclusion criteria can be found in Table 1.

Patients were evaluated through an anonymous electronic questionnaire consisting of closed questions designed to evaluate patient perception of their own degree of autonomy in the management of the disease and the level of knowledge regarding the chronic disease. The survey aimed at achieving a socio-demographic and lifestyle characterization of teenagers on the verge of transition. For the purpose of this study, we used the standards implemented by international investigation questionnaires which were modified to address specific Romanian population issues [22,23,24].

For this study, both patients and their legal guardians signed informed consent forms.

The survey consisted of 20 questions based on chronic disease-specific recommendations regarding specific domains of knowledge identified as important for a successful transition (medication management skills, appointment coordination abilities, health monitoring, medical communication), and 10 questions related to socio-demographic aspects, quality of life, trust in self-managing skills, and future plans. For Section 3.2, Section 3.3, Section 3.4 and Section 3.5, participants were asked to indicate the level of involvement and knowledge they held upon specific aspects and their answers were scored as follows: ‘no, I don’t know how’ (1 point), ‘no, but I want to learn’ (2 points), ‘no, but I am currently learning how to’ (3 points), ‘yes, I have already started to do so’ (4 points), ‘yes, I do this all the time when I am supposed to’ (5 points). Based on participants’ responses, particular and overall scoring levels were calculated.

The questionnaire was evaluated by two separate teams each formed by one pediatric gastroenterology specialist and two gastroenterology and hepatology specialists. Both teams provided their notes and comments on the questionnaire which were later analyzed along with the questionnaire by a third team formed by two Professors of Pediatric Gastroenterology and two Professors of Gastroenterology and Hepatology.

During the assessment of content or face validity, we focused on the clarity of contents, meaning, choosing of words, and item intelligibility. The content of the questionnaire was the subject of 15 interviews with adolescent patients who were only part of the pilot study and were not included in the research. Participants were asked to explain the meaning of each question and to identify any words or concepts that were unfamiliar or unclear to them. Some items were rephrased and simplified based on participant feedback.

Subsequently, the questionnaire was filled in by a sample of 25 patients aged between 14 and 16. Based on their responses, we calculated Cronbach’s alpha coefficient to assess the questionnaire’s internal consistency and reliability. The obtained alpha value of 0.76 allowed the questionnaire to be used in our research. 

Taking into consideration the fact that the questionnaires were completed at home, patients were not precluded from looking up information they felt to be relevant. However, they were encouraged to complete the survey sincerely and were not directly prompted to use alternative informative strategies.

For data processing, input, validation and analysis, we used SPSS 29.0.2.0. Depending on the type of variable, Pearson’s chi-square test was used. Quantitative variables were expressed as numbers and/or percentages. We conducted a Pearson correlation analysis to examine the relationships between various variables from the questionnaire and the overall score. Significant correlations (Sig. 2-tailed) were identified with values less than 0.05. Significance levels were as follows: significant correlations at the 0.01 level (2-tailed) and significant correlation at the 0.05 level (2-tailed).

In our analysis, we used a standard alpha value of 0.05, indicating high significance and ensuring that correlations were not merely due to random sampling error. Despite potentially weak correlations, our study’s robust statistical power, supported by 124 participants, allows for the identification of even subtle effects.

## 3. Results

Participants with chronic digestive diseases recorded in the data base of a tertiary pediatric gastroenterology center and private practices were selected in November 2020. They were treated for inflammatory bowel diseases (ulcerative colitis and Chron’s disease), chronic gastritis, celiac disease, and chronic liver diseases (chronic hepatitis B, chronic hepatitis C, cystic fibrosis with hepatic involvement, liver cirrhosis, Pompe disease with hepatic involvement).

Later, a total of 174 patients were contacted via online forms; 124 (71.26%) participants completed the online survey.

### 3.1. Social and Demographic Characterization

Of the respondents, 73.38% (*n* = 91) lived in rural areas and 26.62% (*n* = 33) in urban areas. Females constituted 59.67% (*n* = 74) of participants. Most of the adolescents attended school (91.93%, *n* = 114), with 6.45% attending only sometimes, and 1.61% had dropped out from school. Future plans included continuing education (73.4%), getting a job (14.5%), and relocating abroad (12.1%). Regarding social activities, 63.70% frequently participated, 25% sometimes, and 11.3% did not participate in any social or sport activities. Family support was strong for 80.65%, and friends’ support was reported by 66.93%. We considered the assessment of cases at risk for school dropout to be important because after the age of 18 the insurance status is lost unless the young adult is enrolled in a form of continuous education (high school, university) or becomes employed.

Regarding general personal healthcare habits, 75% (*n* = 93) of children declared taking care of their health by engaging in physical activities, having a balanced nutrition, and a proper sleep hygiene, while 25% (*n* = 31) of them claimed they only sometimes had such preoccupations.

Participants were evaluated on the significance they placed on independently managing their health. They rated this importance on a scale from 1 (not at all important) to 5 (very important). A total of 61.3% (*n* = 76) of participants indicated that it was very important to manage their own healthcare, while 38.7% (*n* = 48) rated it as important. Additionally, participants’ confidence in their ability to manage their health was assessed on a scale from 1 to 5. The distribution of responses is illustrated in Table 2.

### 3.2. Chronic Disease Medication Management

In the medication management section, patients were queried about their prescription refill practices. A majority of 53.24% (*n* = 66) reported not refilling their prescriptions but expressed a desire to learn how to do it. Additionally, 30.64% (*n* = 38) indicated they simply did not know how to refill prescriptions. Only 6.45% refilled medications independently. Detailed medication knowledge was low (5.64%), but 31.45% expressed desire to learn, and 59.68% were in the process of learning such details. Regarding the management of possible drug-related side effects, 86.3% wanted to learn to deal with them, and 8.06% were actively learning.

The overall and subscale scores by population characteristics can be found in Table 3.

The statistical analysis indicated that adolescents living in urban areas show better managerial skills regarding their medication (*p* < 0.01). There is a positive statistically significant correlation between scores for medication and those for appointment coordination skills, monitoring changes in health and medical communication skills (0.336, *p* < 0.01; 0.451, *p* < 0.01; 0.497, *p* < 0.01). Even though the r value does not indicate a strong relation between the two variables, their correlation is significant due to the large population sample showing data statistical significance (Table 4).

### 3.3. Medical Appointment Coordination

No participants scheduled appointments independently, but 42.74% wanted to learn the process. Only one responder contacted their physician for unusual health changes, while 37.90% wanted to learn how to get in contact with their doctor. Most of the participants followed medical recommendations, with 56.45% reporting some difficulties. Regarding the planning of trips or transportations to medical facilities, 9.67% did not make any plans, 40.33% wanted to learn how, and 50% had begun learning or were already making the necessary arrangements

The statistical analysis shows no correlation between registered scores and gender or environment (Table 4). However, there is a positive statistically significant correlation between scores for appointment making abilities and communication skills (0.336; *p* < 0.01).

### 3.4. Monitoring Healthcare Dynamics

Organizational skill assessment showed that 94.35% lacked a calendar or list of appointments, with 35.48% wanting to learn, and 44.35% already in the process of learning. Upon hospital admission, 44.36% of participants reported providing their medical history, including allergies, on a case-by-case basis about themselves. Meanwhile, 45.96% were in the process of learning how to do it.

A percentage of 61.29% (*n* = 76) reported symptoms to the medical staff on their own. Only 37.90% of participants were being consulted independently, while 31.45% were interested and learning the importance of such practices. Health decisions excluded 83.06% of participants, though 33.87% wanted to learn, and 48.38% were actively learning the process of decision-making.

The statistical analysis showed that teenagers living in urban areas showed better healthcare monitoring skills (0.346; *p* < 0.01). A strong positive relation was found between the capacity to monitor their health dynamics and medical communication skills (0.726; *p* < 0.01).

### 3.5. Medical Communication Skills

A total of 36.30% had started to ask their doctor health-related questions, while 57.25% were in the process of learning how to. Answering medical questions was done independently by 44.35%, with 50% actively learning how to do it. Only 35.49% of participants asked for further clarifications, with 58.87% in the process of learning this. Honesty in following medical advice was reported by 72.58%. Confidence in sharing full medical history including surgeries, allergies, past and current medication was low (4.03%).

There is a negative statistically significant correlation between communication skills and gender (−0.300; *p* < 0.01) suggesting that there may be underlying factors influencing communication abilities based on gender (girls scored higher on this section). A positive statistically significant correlation was found between communication skills and the living environment (0.215; *p* < 0.05) suggesting that teenagers from rural areas have slightly decreased communication skills that could be nurtured during the transition process. Various positive correlations were found between communication skills and the other three sections of the questionnaire: medication management (0.497; *p* < 0.01), appointment coordination (0.342; *p* < 0.01), and monitoring healthcare dynamics (0.726; *p* < 0.01).

### 3.6. Overall Score Analysis

Strong correlations were found between the overall score and all four sections of the questionnaire: chronic disease medication management skills (0.739; *p* < 0.01), appointments coordination abilities (0.635; *p* < 0.01), health monitoring (0.881; *p* < 0.01), and medical communication skills (0.842; *p* < 0.01). A negative correlation was found between gender and the overall score (−0.185; *p* < 0.05) suggesting that, generally, girls tend to have slightly higher scores compared to boys.

A positive correlation was found between the overall score and the living environment (0.335; *p* < 0.01).

**Figure 1:**
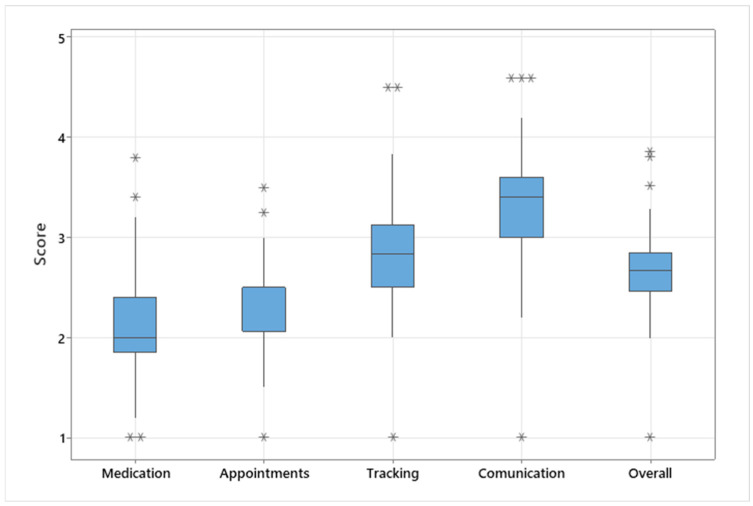
Boxplot of registered scores in the transition readiness assessment questionnaire.

Figure 1 offers a general view of the differences in score distribution. The boxplot evaluates the shape, the central tendency, and the variability of the registered score distribution and offers the possibility to identify extreme or atypical values which can be seen flagged by asterisks. By analyzing the boxplot, we can find that the scores for medication management are the lowest, followed by those for medical appointments and health tracking, while the highest value was obtained for the communication skills section. This suggests that patients may need more intensive or specific medication management training.

## 4. Discussion

Transition is defined as the intentional and planned movement of young adults with chronic conditions from the child-centered to the adult-centered health care system [25]; the goal being to maximize the functioning and developmental potential of adolescents by providing uninterrupted high-quality healthcare services into adulthood. Transfer, on the other hand, is a discrete event that occurs as part of the transition involving the actual transfer from a pediatric to an adult physician [26].

Depending on the needs, the barriers and critical points in the transition process identified by adolescents with chronic diseases, their caregivers and health professionals, and based on the expertise of international guidelines published by specialists, recommendations are proposed for the implementation of gradual and planned transition programs [27]. In order to improve the quality of transition services, it is mandatory to understand the current state of the healthcare transition process and patient preparation and perception regarding the whole process.

International fora have been launching recommendations to improve the transition process. In spite of these efforts, the National Survey of Children with Special Health Care Needs has shown that, in 2001, only 15% of patients had been informed by a medical specialist about the management changes that were about to take place when emerging into adulthood. The same institution conducted another survey in 2006 and found that only a surprising 40% of patients had benefitted from specialized counseling during the transition process [28,29,30].

Transition readiness is a new concept that refers to adolescents’ level of preparation for entering the adult-oriented healthcare system. In order to fully and correctly assess whether a teenager is ready to take responsibility for their own health, one must understand the changes that the adolescent faces during this stage of their lives [31]. In addition, several authors stated that the concept of transition readiness is, in fact, measurable and potentially modifiable before transfer [32,33].

Over the years there appears to have been a lack of consensus regarding the definition of *transition readiness*, therefore it has been difficult to address the issue. Recently, authors have tried to come up with a list of skills that could help understand this ‘umbrella term’: self-management skills, making and keeping appointments, managing one’s medication, noticing the changes in health condition and communicating with their provider [31]. A systematic literature review that analyzed publications related to the psychometric properties of tools used to assess the transition readiness of adolescents with chronic diseases emphasized the fact that most of the available tools cannot be qualitatively evaluated and that only the TRAQ questionnaire (Transition Readiness Assessment Questionnaire) has proven its reliability [22,23,34].

Previous studies report that, in general, the majority of adolescents received limited preparation and education regarding the most important aspects of the transition. However, those who were exposed to some sort of intervention or effort in that matter were more likely to possess motivational skills and to have higher competence skills during the transition [35].

This study evaluated the transition readiness of Romanian adolescents with chronic digestive diseases by examining their self-management awareness, healthcare decision-making involvement, medication knowledge, and communication skills. These aspects are critical for ensuring a smooth transition to adult healthcare and addressing risk factors that may hinder successful long-term outcomes.

Given the recognized challenges adolescents with chronic digestive diseases face during the transition to adult healthcare, this study utilized a validated online questionnaire, completed at home, to comprehensively evaluate key aspects of transition readiness in a comfortable and accessible environment.

In Romania there is no organized transition program, and, currently, the transition consists purely in the transfer of cases and documents which in the vast majority of cases is done by the patient and their family. Consequently, many young adults fail to get proper medical care after the age of 18, leading to treatment interruptions, disease exacerbation, increased morbidity, and mortality. This is, to our knowledge, the first study to assess transition readiness in pediatric Romanian patients emerging into adulthood.

New estimates of transition readiness for adolescent 12 to 17-year-olds with and without special healthcare needs show that 83% of youth with special needs and 86% of youth without special needs do not meet the criteria for successfully initiating the process of transition. In the analysis of these data, the following were taken into account: whether the young person was given time for an individual dialogue with the treating doctor or another doctor during the last medical visit; if the treating doctor or another doctor explained the principle of self-management to the patient and encouraged him/her in this endeavor; whether the changes in healthcare that occurred at the age of 18 had been explained to the young person; and whether they had a consultation with an adult doctor [9,36,37].

A systematic review analyzing 20 years of publications on transition readiness skills identified risk factors which could be potentially modifiable or non-modifiable. According to the authors, a significant part of the potentially modifiable factors seemed to be provider-related and included communication during the transition, the duration of the process, and satisfaction related to the healthcare. Other potentially modifiable factors were related to the age during the assessment, self-efficacy skills, autonomy, medical adherence, and medical appointment management. The identified non-modifiable risk factors were related to demographic characteristics or related to the type of the chronic disease, its duration, the age at diagnosis, the type of followed medication, and the disease activity status [38].

School dropout represents an important risk factor for transition malfunctions, especially since young adults lose their free national health insurance which is normally guaranteed by the state up to the age of 26 years old for people following some form of education. In our study, we identified alarming percentages of adolescents at risk of school dropout (8.06% of participants did not frequently go to school and only attended classes sometimes). This could be a potentially modifiable risk factor through early interventions within the family and through the local social assistance system, with the help of school representatives and psychologists.

Starting with the age of 18 in Romania, the adolescent is considered a young adult, the medical insurance guaranteed by the state for underaged citizens ceases, usually the young adult graduates from high school, and, in general, is either employed or attends university courses. In these cases, the patient benefits continuously from medical insurance by paying the contribution to the health services in the case of employees or through state insurance in the case of students, until the age of 26. In addition, the pediatric age in Romania is 0–18 years of age and when a teenager turns 18 in the 11th or 12th grade, they are no longer evaluated by the pediatric specialist but are provided with the last medical letter whereby they should enter into the adult-oriented healthcare system without any specialized help [39].

However, in poor regions such as the North East Region of Romania, many young adults do not follow the courses of a university, but work outside the law, without an official employment contract, thus being in the position of losing the state insurance in the absence of a direct payment to the National Health Insurance Fund. According to the latest data, the rate of young adults outside the education, employment, or training systems in Romania is between 20 and 25%. Along with strategies to promote continuous education (such as the UNICEF CRIPS program and the ‘Come to school’ national campaign), a series of programs have been proposed in order to encourage employment in this population sector, such as the “Jobs for Youth” initiative, supporting labor mobility within the European Union through the EURES program, and investing in young adults over the European Social Fund [40].

Adolescence is a period of time that comes with a lot of challenges. On one hand, there are the hormonal changes influencing emotional status, self-perception, and relationships; on the other hand, there is the burden of resisting social pressures which is more difficult at this age favoring at-risk behavior, reckless decisions, and rebellious attitudes. Teenagers’ judgment can sometimes be overwhelmed by the desire for new experiences, thrill-seeking, and sexual impulses. Adolescents need to feel independent and explore the limits, break the rules, while still being able to count on their parents for support and protection [41].

Adolescents with chronic conditions may often experience difficulties in building relationships with their peers, which may impair psychological well-being. Data show increasing rates of general prevalence of depression among teenagers (11.3% in 2014); those suffering from chronic diseases appearing to be at a higher risk for mental health issues [42]. Social activities and relationships are particularly at risk mostly due to unpredictable symptom exacerbation, physical changes, daily medication, and the need for constant medical care and supervision [43]. In our study, we identified that 19.35% of surveyed teenagers did not feel endorsed by their families at all times; when referring to friends, 32.25% did not experience support from friends all the time. We acknowledge the importance of the family and peer supportive systems in achieving a good quality of life for these patients in order to encourage healthy behavior, drug adherence, and to diminish disease relapse.

The lack of effective medical and psychological counseling of young adults in the position of making responsible decisions on their own, regarding individual health and chronic disease management, leads to low compliance with treatment and non-compliance regarding appointments for procedures, or abandoning investigation programs and treatment—a number of investigations, such as endoscopic ones, are carried out with total anesthesia or analgesia in pediatric patients, while in services for adults this practice does not exist in every medical center [44].

According to the American Academy of Pediatrics, the American College of Physicians, and the American Academy of Family Physicians—American Society of Internal Medicine, transition readiness should be regularly assessed using an objective measure such as a predefined closed questionnaire, thus allowing both medical providers and patients to identify and grow self-management abilities [45].

Our data support the existing literature data suggesting that adolescents living in urban areas show better managerial skills regarding their medication management (r = 0.332; *p* < 0.01). This suggests that environmental factors, such as access to healthcare resources and education, may influence teenagers’ ability to manage their chronic disease medications effectively. This observation has several practical implications: first, these skills are crucial for ensuring medication adherence and timely adjustments to treatment, which are particularly important for chronic digestive diseases requiring ongoing care. Urban adolescents may benefit from greater exposure to healthcare education initiatives, digital tools, and structured support systems commonly available in urban settings.

The results from our research indicate that teenagers who have better medication management skills also tend to excel in medical appointment coordination (r = 0.336; *p* < 0.01), health monitoring (r = 0.451; *p* < 0.01), and medical communication (r = 0.497; *p* < 0.01). While the correlation coefficient may not indicate a strong relationship between the variables, the correlation is still significant due to the large population sample, indicating statistical significance. These results emphasize the importance of comprehensive healthcare education and support for teenagers with chronic diseases, especially during a delicate process such as the transition.

Workshops focused on improving adolescents’ ability to articulate their health needs and concerns could enhance medication adherence and overall health outcomes.

Regarding medical appointment coordination, the mean scores for teenagers living in rural and urban areas indicate slight differences, with teenagers from urban areas scoring slightly higher on average (2.34 vs. 2.41). However, the statistical analysis shows no correlation between the living environment and medical appointment management skills (*p* = 0.292). This suggests that, regardless of whether they live in rural or urban areas, teenagers exhibit similar levels of proficiency in managing medical appointments. The reason could be related to the fact that parents tend to take full responsibility for their children’s medical care and decision-making process, despite general recommendations to encourage self-management and self-advocacy. Previous studies suggest that achieving independence from both their families and from the pediatric medical team will help bridge the gap towards adult care and will encourage the young adults to make informed decisions about their care [46].

While there is no significant difference in medical appointment management skills based on gender or living environment, there is a clear correlation between appointment coordination abilities and medical communication skills (r = 0.342; *p* < 0.001). These findings highlight the need to tailor transition programs to address gender-specific needs and ensure equitable outcomes. By explicitly connecting these demographic percentages to the study’s findings, the results underscore the importance of context-sensitive approaches to improve transition readiness in diverse populations. Understanding and fostering these skills can empower teenagers to manage effectively their healthcare needs and engage in collaborative decision-making with healthcare providers.

A strong positive correlation was found between the ability to monitor healthcare dynamics and medical communication skills (r = 0.726; *p* < 0.001). This implies that teenagers who are better at observing changes in their health status are also likely to have better communication skills. This correlation suggests that effective communication may be facilitated by a raised awareness of one’s health condition and the changes that may appear.

This relationship may have potential implications for counseling and transition protocols for teenagers, particularly during the transition from pediatric to adult healthcare settings. By emphasizing the importance of monitoring changes in health status and fostering communication skills, the multidisciplinary transition team can better support teenagers in managing their health and effectively communicating their needs during this process, thus improving adolescents’ readiness for the transition. Transition strategies include encouraging the use of digital tools, such as apps or journals, to help adolescents record and share their health information effectively with providers.

The identified gender differences in communication (r = −0.300; *p* < 0.001) skills have also been observed by the specialized literature. Several studies found that adolescent female patients had better health self-management skills such as ordering drug refills and preparing questions for upcoming medical appointments [47,48]. However, other authors observed the fact that boys were more likely to remember specific details such as the date of their last admission and the last endoscopic procedure [49].

Additionally, we observed that teenagers from urban areas tend to have higher communication scores compared to those from rural areas; this may be related to the access to educational resources, increased exposure to diverse social interactions, and differences in community dynamics. Active interventions for this particular population area are mandatory and strategies should be considered involving social workers, primary care physicians, schools, and community organizations in order to integrate medical communication training into existing programs and initiatives. Such strategies could take into consideration telemedicine training programs specifically tailored for rural teenagers, including modules on effective communication skills, understanding medical terminology, and utilizing telehealth platforms. Recent literature reinforces the fact that the improvement in transition communication and skills acquisition has a positive impact on the transition of adolescents with long-term conditions [50].

The overall scores for female responders and male participants indicate that, on average, females scored slightly higher than males. However, the difference between the mean scores is relatively small. The negative correlation between gender and the overall score (r = −0.185; *p* = 0.04) suggests that, in general, females tend to have slightly higher overall scores compared to males.

The positive correlation between the overall score and the living environment (r = 0.335; *p* < 0.001) indicates that teenagers living in urban areas tend to have higher overall scores compared to those living in rural areas. This suggests that environmental factors, such as access to healthcare resources, infrastructure, socioeconimic status, and education may influence teenagers’ overall healthcare and skills in managing their health.

Strong positive correlations were found between the overall score and all four sections of the questionnaire: medication management skills (r = 0.739; *p* < 0.001), appointment coordination abilities (r = 0.342; *p* < 0.001), health monitoring (r = 0.881; *p* < 0.001), and medical communication skills (r = 0.842; *p* < 0.001). This implies that teenagers who score higher in one area are likely to also score higher in other areas. We consider these areas to represent potentially modifiable risk factors to a successful transition process, through active interventions as part of an organized transition process.

Data from our study reinforce the existing information suggesting that adolescents and young adults seem not to be prepared for the transition process. A percentage of 62.10% reported to have never or rarely seen the pediatric gastroenterologist on their own as part of the visit, although international specialized fora recommend that teenagers begin seeing pediatricians independently at least for one part of the visit starting at the age of 14 [51]. Gradual steps, such as the implementation of individual interactions with pediatricians during appointments could foster autonomy and prepare adolescents for adult healthcare responsibilities.

Our study is empowered by the important number of participants and by the fact that it represents the first assessment of transition readiness in a Romanian population. However, there are several limitations that should be mentioned. First, due to the fact that the research took place during the COVID-19 pandemic, we selected the inclusion criteria of age so that it covered a wide range of patients especially because of the social distancing and the limited access to hospitals in the pandemic context. Second, this study is part of a larger research study aimed at improving the transition of teenagers with chronic digestive diseases and, on the pandemic climate, several patients reached the age of 18 years before being able to benefit from the training sessions.

Furthermore, the study was limited to participants from only one region of the country (North-Eastern part of Romania), even though patients belonging to different social backgrounds were included, therefore increasing the potential to generalize the results on wider coverage (national level). Since this research was conducted only in Romania, the results cannot be generalized to other populations because of the socio-economic differences. Inclusion of a larger, more diverse patient database in future research would allow researchers to identify region-specific, disease-specific, and ethnicity-specific risk factors that can prevent a successful transition. What is more, the manner in which the participants were questioned via online survey may seem limited. However, study models on other populations indicate that adolescents who do not attend outpatient appointments with regularity are at a higher risk of poor management and prognosis [52]. Thus, by reaching to our patients via online means, we eliminated this bias and included a representative sample from the entire at-risk population.

In addition, active interventions to address these potentially modifiable risk factors impacting transition readiness are needed and the results of longitudinal studies remain of paramount importance in constructing a successful transition program.

## 5. Conclusions

The importance of a successful transition in children with chronic digestive diseases remains of paramount importance, enhancing the need for systematic organized transition programs. The adolescent is a particularly challenging patient who is in a continuous process of physical, emotional, and cognitive change.

Young adults are increasingly recognized as a vulnerable population not only in terms of risk-taking behavior, but also susceptibility to exacerbation or worsening of chronic conditions, increased skepticism towards allopathic medicine, and reduced referral to healthcare.

The assessment of transition readiness is an important step during the transition process in order to provide personalized care and education. Therefore, the timing of transfer should ideally be founded on individuals’ readiness and not be solely based on a government decision, and should be individualized based on the level of preparation and the amount of time required in order to properly cultivate specific skills.

In our study, we identified disparities in transition readiness among young Romanian adolescents, with a potentially modifiable mechanism to address matters, such as offering a larger time frame for transition and with structured links between pediatric gastroenterologists and adult care. In this setting, there is a crucial role of adult care clinicians in accepting the formation of a partnership with young adults emerging both as a delivery system for the end goal (effective transition—quality of life and superior survival) and as a challenge for education and vocational training.

Notably, the gender differences in communication and the lower transition readiness among adolescents from rural areas highlight the need for tailored interventions. Gender-sensitive transition education could address the specific challenges faced by males and females, while telemedicine or mobile health services may bridge the urban–rural gap in access to care.

Addressing socioeconomic factors such as school dropout, a known risk for reduced healthcare access is essential. Collaborations with schools, family counselors, and community-based programs could improve transition outcomes.

Targeted interventions, such as workshops on effective doctor–patient communication and telemedicine training could empower adolescents to understand their healthcare needs, become familiar with medical terminology, and engage actively in decision-making. Enhanced communication skills would directly improve medication adherence, appointment scheduling, and health monitoring. This would also enable adolescents to recognize and address early warning signs of disease exacerbation, improving long-term outcomes. Other strategies include the implementations of mobile application reminders and pharmacist-led educative sessions.

By focusing on these targeted interventions, healthcare systems can address the weaknesses identified in this study and significantly enhance transition readiness. Beyond the immediate population studied, these findings have broader implications for designing future transition programs in other regions or populations. Programs informed by this research could serve as scalable models, adaptable to diverse healthcare systems and cultural contexts, ensuring that adolescents worldwide can navigate the critical transition to adult healthcare with confidence and competence.

Ultimately, this study provides a roadmap for building comprehensive, evidence-based transition programs that not only improve individual outcomes but also advance global efforts to reduce healthcare disparities and promote lifelong health equity.

Further multicentric prospective research remains mandatory in order to properly assess the transition readiness of adolescents with chronic digestive diseases on a multi-national level, while allowing particular approaches based on national cultural and socioeconomic environment.

As Romania embarks on the crucial journey to develop a standardized transition protocol for children with chronic digestive diseases, the question “Quo vadis?” beckons us towards a future where every young patient is supported through their transition to adult care. By prioritizing comprehensive transition programs, investing in specialized training for healthcare providers, and fostering a collaborative healthcare environment, Romania can pave the way for a healthier future for its young population. The road ahead may be challenging, but the destination promises a brighter, more secure healthcare landscape for all.

## Figures and Tables

**Table 1:** Inclusion and exclusion criteria.

Inclusion Criteria	Exclusion Criteria
Chronic digestive disease clearly defined according to international guidelines [11,12,13,14,15,16,17,18,19,20,21]	Not signing the informed consent forms
Age: 14–17 years old	Age < 14 years old
Signing of the informed consent forms	Not meeting the international criteria for chronic digestive disease

**Table 2:** Distribution of reported confidence levels in managing their own health.

	1	2	3	4	5
How confident do you feel about your ability to manage your own health?	9.67%	43.55%	41.14%	4.84%	0.8%
	n = 12	n = 54	n = 51	n = 6	n = 1

**Table 3:** Overall and subscale scores by population demographic characteristics.

Characteristic	Overall Score Mean and SD			Medication Management			Appointment Coordination			Health Monitoring			Medical Communication		
	Mean	SD	Min–Max	Mean	SD	Min–Max	Mean	SD	Min–Max	Mean	SD	Min–Max	Mean	SD	Min–Max
Gender															
Female (n = 74)	2.71	0.33	1.98–3.85	2.15	0.39	1–3.2	2.39	0.36	1.75–3.5	2.85	0.48	2–4.5	3.45	0.46	2.6–4.6
Male (n = 50)	2.57	0.37	1–3.52	2.06	0.51	1–3.8	2.32	0.39	1–3	2.76	0.43	1–3.66	3.14	0.51	1–4
Environment															
Rural (n = 91)	2.57	0.32	1–3.28	2.02	0.38	1–2.8	2.34	0.38	1–3.33	2.71	0.41	1–3.83	3.26	0.50	1–4.2
Urban (n = 33)	2.84	0.36	2.23–3.85	2.37	0.51	1.6–3.8	2.41	0.34	1.75–3.5	3.06	0.50	2.5–4.5	3.51	0.48	2.6–4.6

**Table 4:** Pearson correlation analysis of the variables from the questionnaire, the overal score, gender, and environment.

Correlations								
		Medication	Appointments	Monitoring	Communication	Overall Score	Gender	Environment
Medication	Pearson Correlation	1	0.336 **	0.451 **	0.497 **	0.739 **	−0.099	0.332 **
	Sig. (2-tailed)		<0.001	<0.001	<0.001	<0.001	0.274	<0.001
	N	124	124	124	124	124	124	124
Appointments	Pearson Correlation	0.336 **	1	0.474 **	0.342 **	0.635 **	−0.088	0.095
	Sig. (2-tailed)	<0.001		<0.001	<0.001	<0.001	0.334	0.292
	N	124	124	124	124	124	124	124
Monitoring	Pearson Correlation	0.451 **	0.474 **	1	0.726 **	0.881 **	−0.097	0.346 **
	Sig. (2-tailed)	<0.001	<0.001		<0.001	<0.001	0.286	<0.001
	N	124	124	124	124	124	124	124
Communication	Pearson Correlation	0.497 **	0.342 **	0.726 **	1	0.842 **	−0.300 **	0.215 *
	Sig. (2-tailed)	<0.001	<0.001	<0.001		<0.001	<0.001	0.017
	N	124	124	124	124	124	124	124
Overall Score	Pearson Correlation	0.739 **	0.635 **	0.881 **	0.842 **	1	−0.185 *	0.335 **
	Sig. (2-tailed)	<0.001	<0.001	<0.001	<0.001		0.040	<0.001
	N	124	124	124	124	124	124	124
Gender	Pearson Correlation	−0.099	−0.088	−0.097	−0.300 **	−0.185 *	1	0.063
	Sig. (2-tailed)	0.274	0.334	0.286	<0.001	0.040		0.487
	N	124	124	124	124	124	124	124
Environment	Pearson Correlation	0.332 **	0.095	0.346 **	0.215 *	0.335 **	0.063	1
	Sig. (2-tailed)	<0.001	0.292	<0.001	0.017	<0.001	0.487	
	N	124	124	124	124	124	124	124

**. Correlation is significant at the 0.01 level (2-tailed). *. Correlation is significant at the 0.05 level (2-tailed).

## Data Availability

The data presented in this study are available on request from the corresponding author due to the large amount of data and privacy concerns.
